# *Leishmania infantum* detection in *Nyssomyia neivai* and dogs in Southern Brazil

**DOI:** 10.1186/s13071-024-06336-z

**Published:** 2024-06-25

**Authors:** Sabrina Fernandes Cardoso, João Victor Costa Guesser, Andressa Alencastre Fuzari Rodrigues, Reginaldo Peçanha Brazil, Luísa Damazio Pitaluga Rona, André Nóbrega Pitaluga

**Affiliations:** 1 Department of Cell Biology, Embryology, and Genetics, Federal University of Santa Catarina (UFSC), Florianópolis, Brazil; 2 Directorate of Epidemiological Surveillance (DIVE), Santa Catarina’s State Health Secretary, Florianópolis, Brazil; 3 Oswaldo Cruz Institute (IOC), FIOCRUZ, Rio de Janeiro, Brazil; 4 National Institute of Science and Technology in Molecular Entomology, National Council for Scientific and Technological Development (INCT-EM, CNPq), Rio de Janeiro, Brazil

**Keywords:** *Leishmania infantum*, *Nyssomyia neivai*, Visceral leishmaniasis

## Abstract

**Background:**

The sand fly *Nyssomyia neivai* is one of the most abundant species in Southern Brazil. It is frequently found in areas that are foci of visceral leishmaniasis in the state of Santa Catarina, caused by *Leishmania infantum*. In this region, the main vector of *L. infantum*, *Lutzomyia longipalpis*, has not been detected. In the absence of *L. longipalpis*, this study aimed to identify the sand fly fauna and diagnose any potential *Leishmania* spp. infection in sand flies and in dogs in a region of Southern Brazil that experienced a recent canine visceral leishmaniasis outbreak.

**Methods:**

This report includes a survey of the sand fly fauna at the Zoonosis Control Center of the Municipality of Tubarão (Santa Catarina, Brazil). Molecular tests were conducted to investigate *Leishmania* spp. natural infection in sand flies using polymerase chain reaction (PCR). In positive females, in addition to morphological identification, molecular analysis through DNA barcoding was performed to determine the sand fly species. Additionally, the dogs were tested for the presence of *Leishmania* spp. using a non-invasive technique for the collection of biological material, to be assessed by PCR.

**Results:**

A total of 3419 sand flies, belonging to five genera, were collected. *Nyssomyia neivai* was the most abundant species (85.8%), followed by *Migonemyia migonei* (13.3%), *Pintomyia fischeri* (0.8%), *Evandromyia edwardsi* (< 0.1%), and species of the genus *Brumptomyia*. (0.1%). Out of the 509 non-engorged females analyzed by PCR, two (0.4%) carried *L. infantum* DNA. The naturally infected females were identified as *Ny. neivai*, in both morphological and molecular analysis. In addition, two out of 47 conjunctival swabs from dogs tested positive for *L. infantum*, yielding an infection rate of 4.2%.

**Conclusions:**

These results confirm the presence of *Ny. neivai* naturally infected with *L. infantum* in an area where dogs were also infected by the parasite, suggesting its potential role as a vector in Southern Brazil.

**Graphical Abstract:**

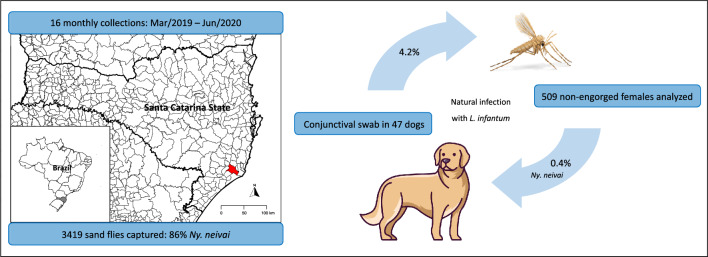

**Supplementary Information:**

The online version contains supplementary material available at 10.1186/s13071-024-06336-z.

## Background

Leishmaniases are diseases caused by protozoa of the genus *Leishmania* (Trypanosomatida: Trypanosomatidae), which can affect both humans and other animals, potentially leading to fatal outcomes. The pathogen transmission occurs through a bite of the sand fly-infected females (Diptera: Psychodidae: Phlebotominae) [1].

Sand flies constitute a group of approximately 1000 known species worldwide, with 530 species having been identified in the Americas [2]. It is estimated that 98 species may serve as potential natural vectors of *Leishmania* spp. [3].

In the Americas, visceral leishmaniasis (VL), the most severe form of the disease, is caused by *Leishmania infantum*. This parasite is mainly transmitted by its primary vector, the sand flies of the *Lutzomyia longipalpis* complex [4, 5]. In Brazil, other sand fly species have been implicated as potential vectors of *L. infantum* in areas where the visceral form of the disease occurs and *Lu. longipalpis* is absent [6, 7]. In the state of Santa Catarina, Brazil, cases of VL affecting both humans and dogs have been reported, yet the conventional vector (*L. longipalpis*) has not been detected [8].

*Nyssomyia neivai*, a sand fly species widely distributed throughout the state of Santa Catarina, is known as the etiologic agent vector of American tegumentary leishmaniasis (ATL) [9]. However, *Ny. neivai* has been found in abundance in regions with human and canine VL cases in Santa Catarina [10].

Based on the VL-positive dog identifications in the Zoonosis Control Center (ZCC) of Tubarão Municipality, Santa Catarina, the present study aimed to investigate potential vectors implicated in the disease transmission and to identify the presence of new cases of canine visceral leishmaniasis (CVL) in the area.

## Methods

**Fig. 1:**
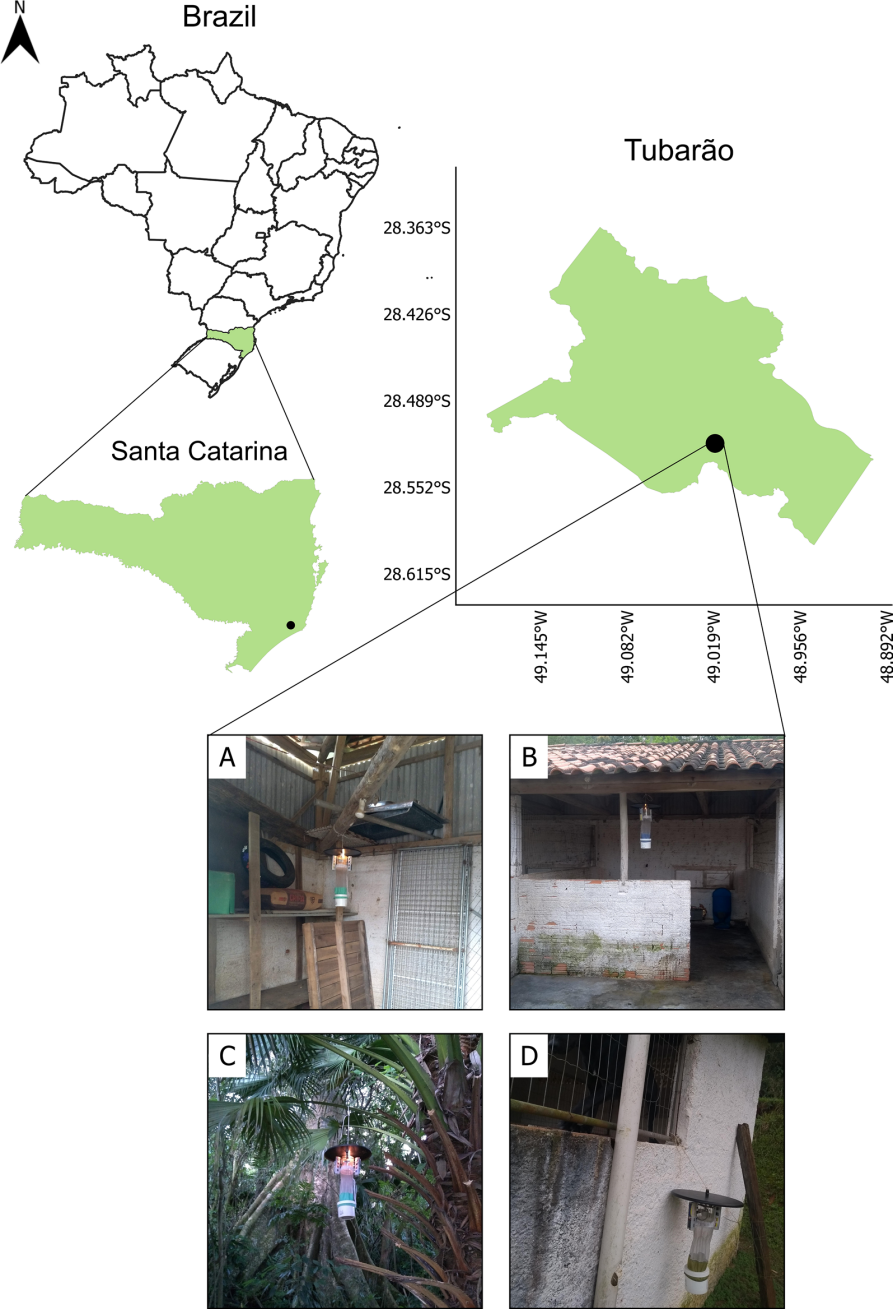
Location of the Zoonosis Control Center (ZCC) of the Tubarão Municipality, located in the south of the state of Santa Catarina, Brazil, and visual representations of the sampling points: **A** trap inside the dogs' stall (point 1); **B** trap in front of the dogs' stall (point 2); **C** traps installed in a forest area (point 3); **D** trap next to the dogs’ stall (point 4)

This study was undertaken at the ZCC of Tubarão (28°31′12.64″S, 49°01′03.09″W), located in the southern region of Santa Catarina (Fig. 1). The institution facility can house around 60 dogs, and there were reports of CVL outbreaks on the premises approximately 3 months before the study began. The investigation was carried out from March 2019 to June 2020.

### Sand fly collections

Collections were performed monthly using four Centers for Disease Control and Prevention (CDC) traps (model 512; John W. Hock, Gainesville, FL, USA) arranged as follows: one inside the dog shelter (point 1), two outside the shelter (points 2 and 4), and one in a nearby forest area (point 3) (Fig. 1). The traps were installed approximately 100 cm above the ground, spaced about 30 m apart, and operated continuously for 12 h, from 6:00 pm to 6:00 am. Once the traps were retrieved, the collected sand flies were sorted into groups of males, engorged females, and non-engorged females, and the specimens were then preserved in 70% ethanol and stored at −20 °C until processing.

### Sand fly identification

The captured sand flies were identified based on morphological characters, using the taxonomic key proposed by Galati [11]. Before being mounted on glass slides, all sand flies underwent a process of clarification, dehydration, and diaphonization of body structures [11, 12]. The full bodies of male sand flies were assembled on glass slides. In engorged females, the head was detached from the rest of the body, while in non-engorged females, the head and the last three abdominal segments were detached from the rest of the body, and then mounted on slides. The remaining fragments of the abdomen and thorax were preserved in 70% ethanol and stored at −20 °C for subsequent analysis.

### Molecular identification of *Leishmania* spp. in sand flies

For molecular identification of *Leishmania* DNA in sand flies, only non-engorged females were considered. This selection aimed to avoid possible *Leishmania* detection originating from an undigested blood meal, focusing instead on identifying potential natural infections. Consequently, the DNA was extracted individually from non-engorged female sand fly body fragments following the protocol established by Jowett [13].

Samples were pooled, each containing genetic material of up to 10 individuals for initial molecular screening. A polymerase chain reaction (PCR) was conducted targeting a fragment of 300–350 base pairs (bp) of the ITS1 region of trypanosomatid ribosomal DNA (rDNA), using the primers and amplification conditions described by El-Tai et al. [14] and Schönian et al. [15]. This sequence varies in size and nucleotide sequence among *Leishmania* spp., facilitating their differentiation [15]. When a positive pool was identified, the original 10 female samples were submitted to an individual analysis through a second PCR reaction under the same conditions. Therefore, the actual number of infected sand flies was identified, thus helping to determine the natural infection rate and the vector identification.

The amplified fragments resulting from the individual sample PCR reactions were purified using the QIAquick^®^ PCR Purification Kit (Qiagen), according to the manufacturer's instructions. The purified fragments were submitted to a Sanger sequencing at the Fiocruz Sequencing Unit (RPT1A, Rio de Janeiro, Brazil) for *Leishmania* spp. identification. Sequencing was carried out in both directions in an ABI Prism 3730 DNA sequencer using the ABI Prism BigDye Terminator Cycle Sequencing Ready Reaction Kit (Applied Biosystems). The sequences were aligned using the Geneious Prime program to obtain consensus sequences followed by Basic Local Alignment Search Tool nucleotide (BLASTN) analysis. The identified sequences were submitted to GenBank (www.ncbi.nlm.nih.gov) under accession numbers OP718537 and OP718538.

### Sand fly molecular identification

In addition to morphological characterization, *Leishmania* spp. positive sand flies underwent molecular identification through DNA barcoding. The primers used and the reaction conditions were those described by Folmer et al. [16] and Pinto et al. [17], amplifying a ~700-bp fragment of the cytochrome *c* oxidase subunit I (*COI*) mitochondrial gene, suitable for invertebrate metazoans, including sand flies. Purified PCR products were sequenced and analyzed following the procedure outlined above. The resulting sequences have been deposited in GenBank under the following accession numbers: OP719771, OP719772, OP719773, and OP719774.

### Dog sample collection

Biological samples were collected from 47 dogs sheltered at the ZCC using the non-invasive conjunctival swab technique [18]. During the procedure, the lower eyelid was slightly separated so that the cotton swab could be rubbed on the ocular conjunctiva to collect cells from the right eye of each dog. Only the swab tips were transferred to sterile tubes containing 200 μl of extraction buffer-SQ solution [13] and stored at −20 °C until analysis.

### Molecular diagnosis of *Leishmania* spp. in dogs

The DNA was individually extracted from each swab according to the protocol established by Jowett [13]. PCR was performed to target the ITS1 region, as described above [14, 15], and the amplicons were sequenced to identify dogs that were positive for *Leishmania* spp., as indicated previously. The resulting sequences were submitted to GenBank under the accession numbers OP724554 and OP724555.

## Results and discussion

### Sand fly abundance

During the study period, 16 collections were performed, totaling 3419 sand flies captured. In most collections (15 out of 16), males outnumbered females (2478 males vs. 941 females) (Table S1), yielding a male/female ratio of 2.63, consistent with previous studies [19–21]. The prevalence of males can be attributed to the formation of male swarms near the vertebrate host during mating, wherein sexual pheromones, coupled with the vibrations from males’ wings, attract females for copulation [22, 23].

In addition, the sand fly distribution varied notably, with the greatest abundance occurring in March and April 2019 (Table S1). These two collections accounted for more than half of all captured sand flies, comprising 1861 out of 3419 specimens. Incidentally, this coincided with the influence of the El Niño climate phenomenon [24], which can affect sand fly seasonality due to higher temperatures [25]. Conversely, no sand flies were collected in May 2019 due to severe storms, which were also linked to El Niño in the region.

Among the four collection stations, point 1 yielded 81.5% of the specimens, followed by point 2 (11.9%), point 4 (5%), and point 3 (1.6%) (Fig. 1, Table 1). The high number of specimens at point 1 can be attributed to its location directly above the dogs' sleeping area, providing abundant food, shelter, and favorable conditions for insect reproduction. Traps at points 2 and 4 were exposed to weather conditions outside the shelter, while the trap at point 3 was in a forest area with relatively lower availability of blood meals for sand flies.

**Table 1:** Sand fly species per sampling point, collected with CDC light traps from March 2019 to June 2020, in Tubarão, Santa Catarina, Brazil

Species	Sampling points									
	Point 1	%	Point 2	%	Point 3	%	Point 4	%	Total	%
*Ny. neivai*	2428	87.2	319	78.6	34	63.0	151	87.3	2932	85.8
*Mg. migonei*	341	12.2	81	19.9	12	22.2	21	12.1	455	13.3
*Pi. fischeri*	16	0.6	5	1.2	6	11.1	1	0.6	28	0.8
*Brumptomyia* spp.	0	0.0	1	0.3	2	3.7	0	0.0	3	0.1
*Ev. edwardsi*	1	< 0.1	0	0.0	0	0.0	0	0.0	1	< 0.1
Total	2786	81.5	406	11.9	54	1.6	173	5	3419	100.00

### Sand fly diversity

Five genera and four species of sand flies were identified based on their morphology. The most abundant was *Ny. neivai* (85.8%), followed by *Migonemyia migonei* (13.3%), *Pintomyia fischeri* (0.8%), *Brumptomyia* spp. (0.1%), and *Evandromyia edwardsi* (< 0.1%) (Table 2).

**Table 2:** Number of sand flies collected and species

Sand fly species	Males		Females				Total	%
	*n*	%	Engorged	Non-engorged	*n*	%		
*Ny. neivai*	2209	89.2	286	437	723	76.8	2932	85.8
*Mg. migonei*	251	10.1	136	68	204	21.7	455	13.3
*Pi. fischeri*	15	0.6	9	4	13	1.4	28	0.8
*Brumptomyia* spp.	3	0.1	0	0	0	0.0	3	0.1
*Ev. edwardsi*	0	0	1	0	1	0.1	1	< 0.1
Total	2478	100	432	509	941	100	3419	100

*Nyssomyia neivai* was the predominant species found in this study, which is consistent with its identification in studies of sand fly fauna in Southern Brazil [9, 26–28]. This species is prevalent in colder and drier Brazilian regions, thriving in South, Southeast, and Midwest Brazil [29]. It is an important ATL vector, and its infection with *L. infantum* has been previously documented in Santa Catarina, Paraná, and Minas Gerais states [19, 28, 30]. Also, *Ny*. *neivai* predominated in all sampling points due to its adaptability to altered environments, particularly animal shelters and closed spaces (Table 1). The ZCC, located in a human-altered native forest fragment, offers ideal conditions for *Ny*. *neivai* proliferation, with shaded, moist, organic-rich soil and a high dog density [26, 28].

Although captured in smaller quantities, *Mg. migonei* and *Pi. fischeri* are also epidemiologically important in *Leishmania* transmission. *Migonemyia migonei* is highly susceptible to *L. infantum* [7] and has been found infected in specific VL foci [31]. Similarly, *Pi. fischeri* is considered a potential VL vector due to its high attractiveness to dogs and susceptibility to *L. infantum* infection [32, 33].

Sand flies belonging to *Brumptomyia* spp. and *Ev. edwardsi* were found in very low numbers at the sampled points. *Evandromyia edwardsi*, a solitary species, has been considered a potential vector of ATL in wild environments [34]. However, sand flies of the genus *Brumptomyia* have no role in leishmaniasis transmission; they mainly feed on armadillos, suggesting their presence in the area [35].

These results are aligned with a previous study that detected the presence of these sand fly species in the state of Santa Catarina [19] and contribute to a deeper knowledge of the state’s sand fly fauna and canine leishmaniasis transmission, which are limited.

### *Leishmania* DNA detection in sand flies

Out of the 941 female sand flies captured, 432 were engorged, and 509 were non-engorged (Table 2). Engorged females were only collected at point 1 (Fig. 1), where the CDC light trap was installed inside the canine shelter. Given the photophilic behavior of these insects and the confinement of dogs, facilitating blood-feeding and sand fly aggregation, it was expected that females post-blood meal would be captured at this collection point [9, 19, 28, 31, 36].

**Fig. 2:**
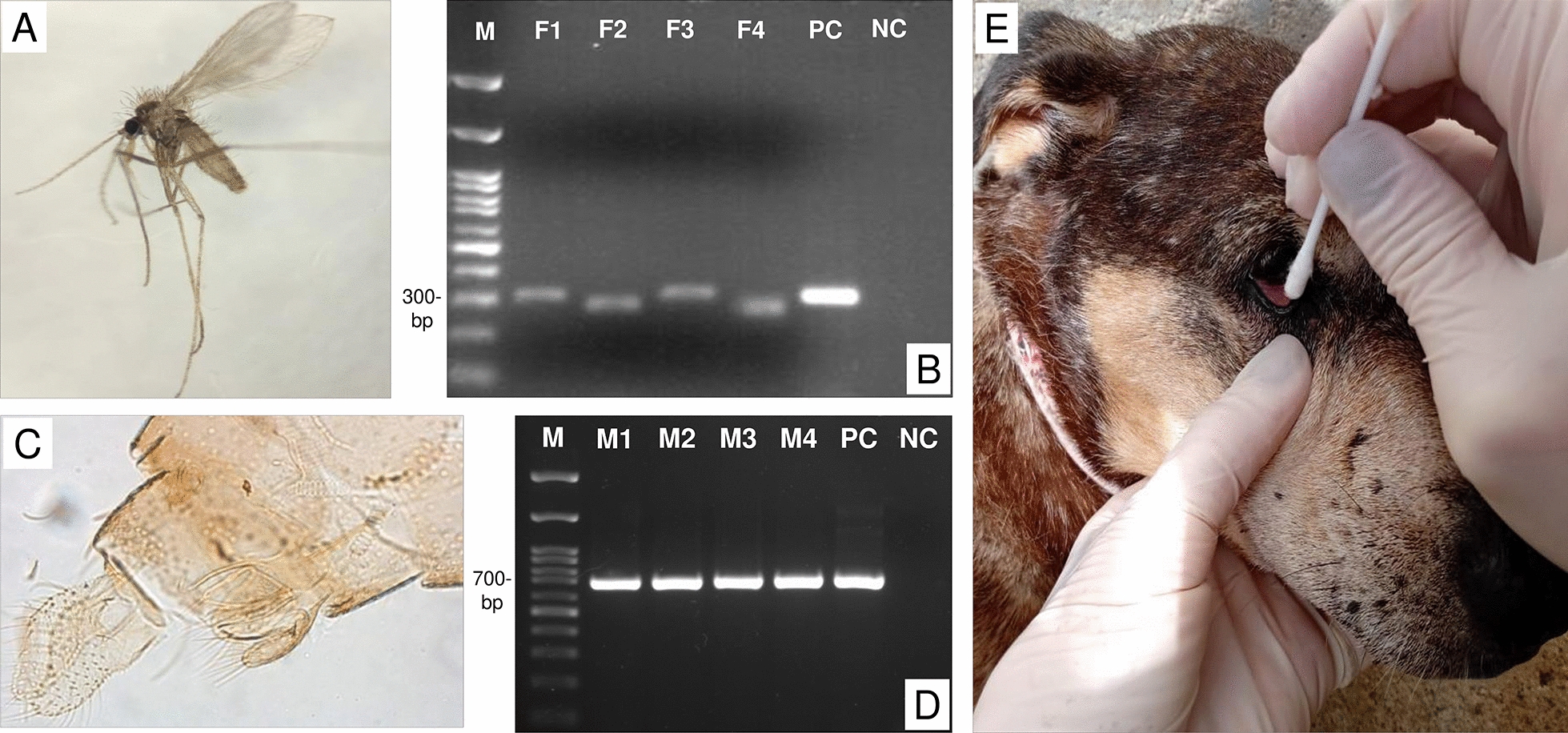
Dashboard displaying a summary of the survey results:** A** non-engorged female adult sand fly. **B** Polymerase chain reaction (PCR) for detecting natural *Leishmania* spp. infection, using primers targeting the ITS1 region of rDNA, which amplifies within the range of 300 to 350 base pairs (bp), depending on the *Leishmania* species. Line M: molecular marker (100 bp), lines F1–F4: female sand flies (*Ny. neivai*) positively identified for trypanosomatids, lines F1 and F3: *Ny. neivai* females positive for *L. infantum*, line PC: positive control using *L. infantum* DNA, line NC: negative control, without DNA, using water instead. **C** Female sand fly specimens mounted on glass slides, highlighting morphological features of *Ny. neivai* spermatheca. **D** PCR conducted for molecular identification of *Ny. neivai* using DNA barcoding. Line M: molecular marker (100 bp), lines M1–M4: female *Ny. neivai* sand flies, line PC: positive control using *Ny. neivai* DNA, line NC: negative control, without DNA, using water instead. **E** Collection of biological material from dogs for *Leishmania* spp. infection detection, employing the non-invasive conjunctival swab technique

Only non-engorged females—that is, 54.1% of collected females—were tested for *Leishmania* spp. infection (Fig. 2A). Out of 53 pools analyzed by PCR, four tested positive for trypanosomatid DNA. Individual DNA analysis of insects within these four pools subsequently detected four specimens positive for trypanosomatids by PCR (Fig. 2B). Sequencing data identified *L. infantum* as the causative agent of natural infection in two out of four sand flies (Fig. 2B: F1 and F3), resulting in a natural infection rate of 0.4%. This infection rate is consistent with previous reports that indicated *Leishmania*-infected sand flies ranging from 0.1 to 1.2% [37].

The four sand flies detected with trypanosomatids were identified as *Ny. neivai*, confirmed through morphological and molecular analyses (Fig. 2C, D). This finding supports previous studies indicating *Ny. neivai*'s natural infection in Brazil with *Leishmania braziliensis* [26, 38] and *L. infantum* [19, 28, 30].

These results present the first DNA barcode sequences of *Ny. neivai* originating from Brazil, ranging in length from 554 to 672 bp. This is an important step towards a precise *Leishmania* vector sand fly species identification in South America, since GenBank currently holds shorter sequences only from Argentina samples (543 bp) (accession numbers MN857519 up to MN857540) [39].

### *Leishmania* DNA detection in dogs

A total of 47 dogs from the Tubarão municipality ZCC were tested for *Leishmania* spp. infection using a non-invasive method for sample collection, the conjunctival swab (Fig. 2E). This simple and efficient technique, combined with ITS1 PCR amplification, has been used for detecting *L. infantum* DNA in asymptomatic dogs [40, 41]. The dogs examined were mixed breed and were asymptomatic for leishmaniasis throughout the sampling period.

The individual DNA analysis of 47 dogs revealed four specimens positive for trypanosomatids through PCR. Subsequent sequencing data identified *L. infantum* as the causative agent of natural infection in two out of the four animals, yielding a natural infection rate of 4.2%. These same 47 dogs were retested by the Santa Catarina Central Public Health Laboratory (LACEN/SC), using the Dual Path Platform rapid test (TR DPP^®^) test (Biomanguinhos/Fiocruz, Brazil, 2011) with serum samples, confirming these findings.

### The leishmaniasis profile in the state of Santa Catarina (Southern Brazil)

In 2010, the state of Santa Catarina reported its first autochthonous case of CVL in the capital city of Florianópolis. Since then, over 1000 cases of infected dogs have been reported across the state. During this period, only Florianópolis confirmed locally acquired cases. Between 2017 and 2023, five autochthonous human VL cases were recorded in Florianópolis, with no reports elsewhere in the state [10]. Interestingly, in these areas with VL cases in both humans and dogs, *Ny. neivai* was prevalent, while the primary vector, *Lu. longipalpis*, was absent [10, 42]. However, a combination of capture methods such as manual capture with a Castro aspirator and CDC light traps, as used by Silva et al. [43], is essential to confirm the absence of *Lu. longipalpis*.

In 2018, 10 cases of CVL were identified at the ZCC of Tubarão. Additionally, two more cases were found in 2020 during this study. Our findings also revealed a high prevalence of *Ny. neivai*, as well as natural infection by *L. infantum*, which was also observed in infected dogs within the study area. The *L. infantum* ITS1 sequence profile was identical in all four samples, two positive sand flies and two positive dogs. This suggests that *Ny. neivai* is attracted to feed on dogs [44] and may play a role in supporting parasite infection after blood digestion, potentially facilitating the transmission of *L. infantum*. *Nyssomyia neivai* is opportunistic and eclectic, feeding on both humans and dogs [44]. While dogs may not be *Ny. neivai*'s preferred blood meal source when other hosts are present, females adjust their feeding patterns based on host availability [44]. This was evident in this study, where engorged *Ny. neivai* females were predominantly captured within the dog shelter (Fig. 1: point 1).

The vectorial permissiveness of *Ny. neivai* for *L. infantum* remains uncertain despite its competence in transmitting *L. braziliensis* [45]. Further investigations are required to confirm its role as an *L. infantum* vector. This involves analyzing further parameters beyond those covered in this study, considering factors like anthropophilic behavior, vector competence, susceptibility to infection [46], and critical aspects of the parasite–vector interaction [47]. Also, confirming the vector capacity of sand flies in transmitting *Leishmania* typically requires dissecting the insect's digestive tract to visualize the protozoan [48]. However, the detection of *L. infantum* DNA in sand flies and dogs in an area loaded with *Ny. neivai* and CVL cases suggests a potential association of this species with *L. infantum* transmission in Southern Brazil.

## Conclusions

In this study, we provide the first evidence of *L. infantum* infecting both *Ny. neivai* and dogs within the same area in Brazil. Considering the absence of *Lu. longipalpis* in the state of Santa Catarina so far, our data suggest that *Ny. neivai* might act as a VL vector transmitting *L. infantum* in that region. Furthermore, we emphasize the importance of using DNA barcoding as a tool to support fast and numerous sand fly identification.

### Supplementary Information

Supplementary material 1: Table S1. Number and percentages of sand flies divided by sex, collected with CDC type light traps from March 2019 to June 2020, in Tubarão, Santa Catarina, Brazil. ♂ = male; ♀ = female.

## Data Availability

The datasets generated during the current study are available in the GenBank database under the accession numbers OP718537, OP718538, OP719771, OP719772, OP719773, OP719774, OP724554, and OP724555.

## Abbreviations

PCR: Polymerase chain reaction
VL: Visceral leishmaniasis
ATL: American tegumentary leishmaniasis
ZCC: Zoonosis Control Center
CVL: Canine visceral leishmaniasis
bp: Base pairs
