# Supplementary material for: Leishmania infantum detection in Nyssomyia neivai and dogs in Southern Brazil
Source: Parasit Vectors. 2024 Jun 25;17:269. doi: 10.1186/s13071-024-06336-z (PMC11200822; doi:10.1186/s13071-024-06336-z)
Supplement: Supplementary file 1 — Supplementary material 1: Table S1. Number and percentages of sand flies divided by sex, collected with CDC type light traps from March 2019 to June 2020, in Tubarão, Santa Catarina, Brazil. ♂ = male; ♀ = female. [file 13071_2024_6336_MOESM1_ESM.docx]

| **Date** | **Male (n)** | **♂ (%)** | **Female (n)** | **♀ (%)** | **Total** | **%** |
| --- | --- | --- | --- | --- | --- | --- |
| Mar/19 | 355 | 75.05 | 118 | 24.95 | 473 | 13.83 |
| Apr/19 | 1095 | 78.89 | 293 | 21.11 | 1388 | 40.60 |
| May/19 | 0 | 0.0 | 0 | 0.0 | 0 | 0.0 |
| Jun/19 | 110 | 61.80 | 68 | 38.20 | 178 | 5.21 |
| Jul/19 | 44 | 64.71 | 24 | 35.29 | 68 | 1.99 |
| Aug/19 | 1 | 100.00 | 0 | 0,0 | 1 | 0.03 |
| Sep/19 | 36 | 66.67 | 18 | 33.33 | 54 | 1.58 |
| Oct/19 | 75 | 64.10 | 42 | 35.90 | 117 | 3.42 |
| Nov/19 | 210 | 69.54 | 92 | 30.46 | 302 | 8.83 |
| Dec/19 | 169 | 75.45 | 55 | 24.55 | 224 | 6.55 |
| Jan/20 | 36 | 53.73 | 31 | 46.27 | 67 | 1.96 |
| Feb20 | 139 | 66.51 | 70 | 33.49 | 209 | 6.11 |
| Mar/20 | 116 | 64.44 | 64 | 35.56 | 180 | 5.26 |
| Apr/20 | 76 | 64.41 | 42 | 35.59 | 118 | 3.45 |
| May/20 | 8 | 61.54 | 5 | 38.46 | 13 | 0.38 |
| Jun/20 | 8 | 29.63 | 19 | 70.37 | 27 | 0.80 |
| Total | 2478 | 72,5 | 941 | 27,5 | 3419 | 100.00 |
